# Utility of CA-125 for diagnosis and prognosis of breast cancer: a systematic review

**DOI:** 10.37349/etat.2025.1002316

**Published:** 2025-05-15

**Authors:** Zohre Momenimovahed, Afrooz Mazidimoradi, Leila Allahqoli, Zohre Khalajinia, Hamid Salehiniya, Ibrahim Alkatout

**Affiliations:** IRCCS Istituto Romagnolo per lo Studio dei Tumori (IRST) “Dino Amadori”, Italy; ^1^Reproductive Health Department, Qom University of Medical Sciences, Qom 3715835155, Iran; ^2^Epidemiology Department, Shiraz University of Medical Sciences, Shiraz 7134845794, Iran; ^3^Midwifery Department, Ministry of Health and Medical Education, Tehran 1467664961, Iran; ^4^Department of Midwifery, Qom University of Medical Sciences, Qom 3715835155, Iran; ^5^Department of Epidemiology and Biostatistics, School of Health, Social Determinants of Health Research Center, Birjand University of Medical Sciences, Birjand 9717853577, Iran; ^6^Department of Obstetrics and Gynecology, University Hospitals Schleswig-Holstein, Campus Kiel, 24105 Kiel, Germany

**Keywords:** CA-125, prediction, screening, diagnosis, breast cancer

## Abstract

**Background:**

Different tumor markers are utilized in the assessment of breast cancer. The function of these markers in assessing, tracking, and following up on breast cancer has drawn the interest of numerous researchers. Nonetheless, contradictory findings from research continue to raise questions regarding their effectiveness. Consequently, this research was carried out to evaluate the efficacy of carbohydrate antigen-125 (CA-125) in the treatment of breast cancer.

**Methods:**

A thorough investigation was performed in the PubMed, Scopus, and Web of Science databases utilizing relevant keywords: CA-125, breast cancer, screening and diagnosis, and Mesh to locate articles published before August 2023 without any time limitations. The analysis included observational studies in English pertinent to the study’s objective, while review articles, case reports, editor letters, comments, and other reports were not considered. Articles were sought, examined, included, and evaluated according to the guidelines of the Preferred Reporting Items for Systematic Reviews and Meta-analyses. The EndNote X9 program has been utilized for item management. The review included articles that investigated the predictive function of CA-125 in the screening, diagnosis, and anticipation for the early and proper detection of breast cancer.

**Results:**

In the initial search, 1,475 articles were obtained. After screening and eligibility assessment, 33 studies were reviewed. Based on the findings of the studies, CA-125 can play a role in the diagnosis of breast cancer, its type and stage, early detection of recurrence and metastasis, treatment efficiency, prognosis, and survival rate.

**Discussion:**

The role of CA-125 as a biomarker for early detection, staging, and monitoring of recurrence and metastasis in breast cancer is still uncertain and needs additional research.

## Introduction

Breast cancer presents a substantial and urgent global health challenge, affecting millions of women and demonstrating increasing prevalence and mortality rates [1, 2]. Contrary to previous beliefs that it predominantly impacted developed nations, the majority of cases and two-thirds of deaths now occur in less developed countries [1]. This shift highlights the need for enhanced efforts in addressing breast cancer on a global scale, particularly in resource-limited settings.

Fortunately, advancements in early detection and treatment have led to an increase in the 5-year survival rate for breast cancer patients worldwide. However, it is disheartening that the mortality rate for this disease has also seen an increase during this period [3]. Despite undergoing surgery and radiotherapy, 20–30% of women with breast cancer develop distant metastases [4]. While the prognosis for many women with metastatic breast cancer is grim, there are still individuals who defy the odds and overcome the disease [5].

The diagnosis and treatment of breast cancer have grown more intricate, creating difficulties at every stage of care. Restricted resources additionally complicate the situation and could jeopardize service quality. Even with some benefits from digital progress, there is still a demand for better solutions [6]. Traditional diagnostic tests, such as chest X-rays, liver ultrasounds, bone X-rays, and CT scans, are still commonly used to rule out metastasis. However, these tests can be costly, require specialized expertise, and rely on high-quality equipment. These factors can pose obstacles and lead to treatment delays [7, 8]. Non-invasive biomarkers such as serum tumor markers play a crucial role in diagnosing and monitoring the effectiveness of cancer treatments. They offer numerous advantages, including accurate and reproducible results, making them an ideal option for diagnosing and monitoring malignant tumors [9, 10].

A variety of tumor markers, such as carcinoembryonic antigen (CEA), carbohydrate antigen 125 or cancer antigen 125 (CA-125), and CA15-3, play a crucial role in the evaluation and monitoring of breast cancer. Recent research has shown a growing interest in comprehending the significance of these markers in the ongoing assessment and follow-up care of breast cancer patients [11]. However, there have been conflicting study findings regarding the effectiveness of CA-125 in breast cancer screening and diagnosis, which has sparked a need for a comprehensive review of its predictive role. This study aims to thoroughly investigate the practicality of utilizing CA-125 for both the diagnosis and prognosis of breast cancer.

## Materials and methods

### Search strategy

We conducted a systematic review using the Preferred Reporting Items for Systematic Reviews and Meta-Analyses (PRISMA) guidelines. In August 2023, we extensively searched PubMed/MEDLINE, Scopus, and Web of Science databases using keywords such as breast cancer/carcinoma/neoplasm/tumor in combination with diagnosis, marker, biomarker, screening, detection, CA 125, CA125, CA-125, CA 125 antigen, and cancer antigen 125.

### Inclusion and exclusion criteria

This review only includes observational studies published in English that explore the significance of CA-125 in screening, diagnosing, and predicting early and accurate detection of breast cancer. It excludes review studies, case reports, letters to editors, conference presentations, non-full-text articles, commentaries, and reports.

### Screening and selection of studies

The retrieved articles were entered into EndNote X9 software and any duplicates were removed. Two authors independently assessed the titles and abstracts to find relevant articles for the review’s aim. In case they were unable to reach an agreement, a third author was available for consultation. Articles examining the prediction role of CA-125 in screening, diagnosis, and prediction for early and appropriate detection of breast cancer were eligible for analysis.

### Data synthesis and data extraction

In our analysis, we focused on describing the outcomes of each review. We presented the results in a table format, along with an abstract. To gather the necessary data, we used a checklist to extract information, including the author, publication year, country of study, inclusion and exclusion criteria, sample size and type, and main result. We categorized this information and presented it in a separate table.

## Results

### Selection of the studies

**Figure 1:**
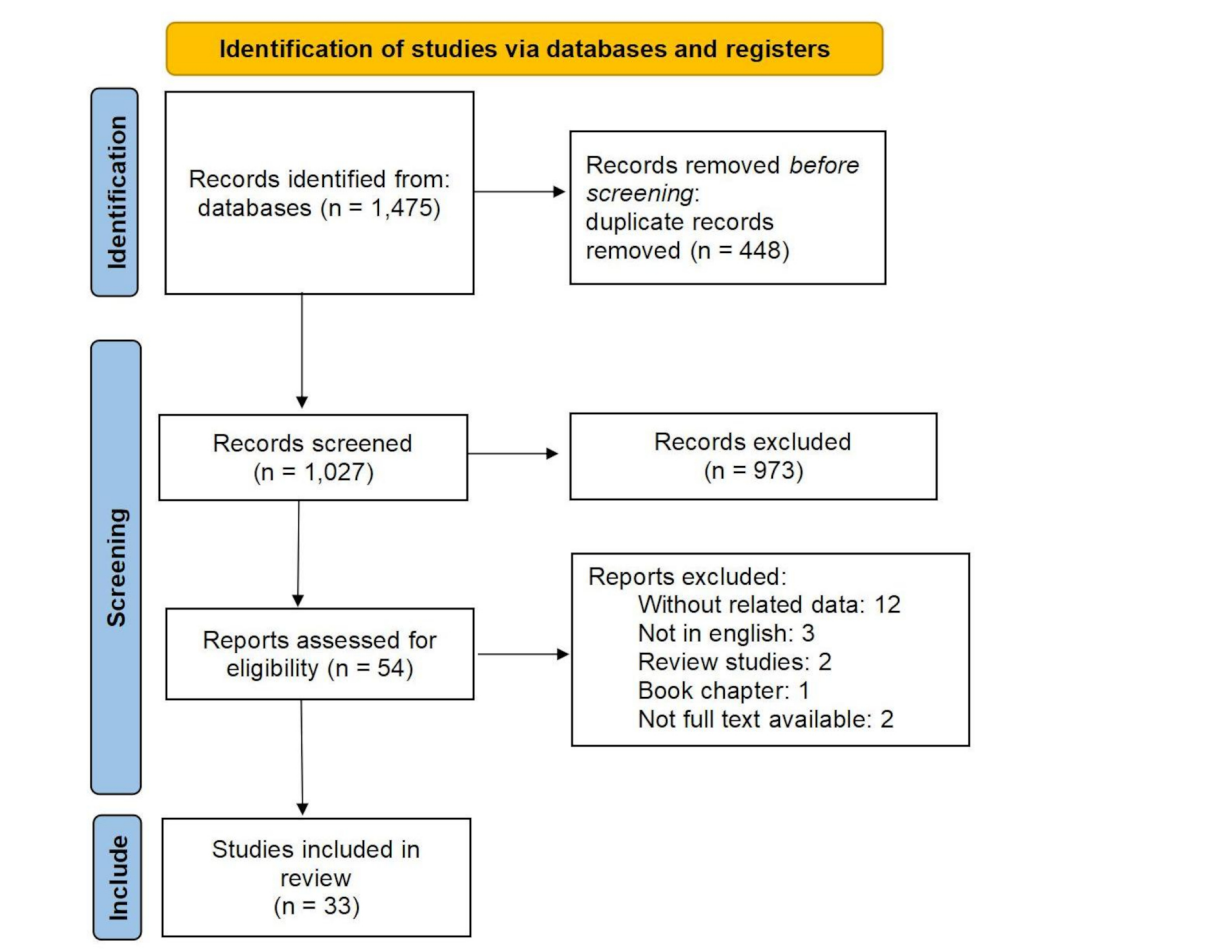
**The process of screening and selecting relevant studies was based on the Preferred Reporting Items for Systematic Reviews and Meta-Analyses.** Adapted from [12], CC BY

A total of 1,475 studies from various databases were initially compiled in Endnote software. After eliminating duplicate records (448 studies), 1,027 studies were chosen for assessment. Upon reviewing titles and abstracts, 973 studies were found to be inconsistent with the objectives of the current study and were subsequently excluded. The complete text of 54 studies was carefully examined, resulting in the exclusion of 21 studies for specific reasons (without related data: 12, not in English: 3, review studies: 2, book chapter: 1, no full text available: 2, laboratory studies: 1). Ultimately, 33 studies were deemed suitable for inclusion in the review as shown in Figure 1. PRISMA 2020 flow diagram for new systematic reviews was used in this study [12].

### Characteristics of included studies

Thirty-three articles were included in the study between 2001 and 2023. The majority of studies (66%) were conducted in China. The sample size ranged from 5–10,836 patients in varied study designs (4 case-control studies, 5 prospective follow-up studies, 14 retrospective studies, 2 cross-sectional studies, 1 nested case-control study, and 6 no mention of the study type). In this systematic review, a comprehensive analysis was conducted on a total of 21,324 samples as shown in Table 1.

**Table 1:** Characteristics of included studies

**First author (year)**	**Study design**	**Location**	**Inclusion criteria (original study)**	**Exclusion criteria (original study)**	**Sample size**	**Sample type**	**Main result**
Agha-Hosseini, et al. (2009) [13]	Case-control study	Iran	Case: patients who are candidates for surgery Control: healthy women	-	Healthy women: 25 Untreated breast cancer: 24 Treated breast cancer: 23	Serum and unstimulated whole saliva	The mean saliva and serum cancer antigen 125 (CA-125) levels were significantly higher in untreated cancer women compared to healthy and treated groups
Fang et al. (2017) [14]	Case-control study	China	Invasive breast cancer	-	Invasive breast cancer: 151 Control: 180	Serum	High preoperative CA-125 levels may reflect tumor burden and are associated with aggressive molecular subtype
Gioia et al. (2016) [15]	Prospective follow-up study	Germany	End of the adjuvant therapy (chemotherapy and/or radiotherapy) First treatment with a therapeutic approach	Patients with a history of Metastatic disease (lymph node and organ metastases) Patients under palliative treatment	Metastatic group: 47 Control group: 48	Serum	The assessment of CA-125 in combination with carcinoembryonic antigen (CEA) and CA 15-3 can be a useful tool in follow-up
Norum et al. (2001) [16]	Retrospective study	Norway	Patients examined at least three times	-	221	Serum	Increased CA-125 was associated with metastasis in or near the pleura, and in stage IV breast cancer, it was related to poor prognosis
Zhang et al. (2021) [17]	Prospective study	China	Not prohibiting imaging examinations Absence of other malignant tumors Acceptance of neoadjuvant therapy	Incomplete clinical data The presence of inflammation and other diseases affecting the results of the research Failure to cooperate with clinical follow-up	65	Serum	Serum levels of CA-125 are useful for the evaluation of the impact of neoadjuvant chemotherapy on breast cancer patients
Chen et al. (2017) [18]	Retrospective study	China	-	Bone metabolic diseases Kidney failure Eating disorders Another primary malignancy Breast patients with other distant organ metastases such as lung, liver, and brain metastases	2,133	Serum	Axillary lymph node metastases and the concentrations of CA-125, CA-153, alkaline phosphatase (ALP), and hemoglobin were the independent risk factors for bone metastases in patients with breast cancer
Gaughran et al. (2020) [19]	Retrospective study	Australia	-	Patients without three or more tumor markers within 4 weeks of diagnosis Patients without four tumor markers at 3 months after diagnosis Patients without imaging at diagnosis Patients with other concurrent malignancies	193	Serum	Increased CA-125 was significantly associated with pleural/peritoneal metastases
Ju et al. (2016) [20]	Case-control study	China	-	-	Initial diagnostic:47 Recurrent:44 Healthy control: 43	Serum	Serum CA-125 levels in recurrent breast cancer patients were higher than in initial diagnostic patients
Yerushalmi et al. (2012) [21]	Retrospective study	Canada	Visiting the patient in stage M 1 Distant recurrence in later stages	Previous, synchronous, or subsequent invasive or in situ cancer of any site other than nonmelanoma skin	810	-	Elevation of CA-125 was documented in the majority of patients with metastatic breast cancer
Ma et al. (2022) [22]	Retrospective study	China	18-75 years old ECOG score of 0-1 Er, pr positive and human epidermal growth factor receptor 2 (HER-2) negative IV stage	Acute or chronic inflammation Breast cancer in males HER-2 positive and triple-negative breast cancer	130	Serum	The high CA-125 related to worse overall survival than the low CA-125 group
Lian et al. (2019) [23]	Retrospective study	China	No history of cancer Complete medical record Performing serum tumor markers two weeks before surgery No history of radiotherapy/chemotherapy/endocrine therapy before surgery	Unknown TNM stage Breast cancers in male Other cancer Patients with stage IV disease at diagnosis	Breast cancer: 804 Healthy women: 305	Serum	In comparison with the healthy volunteer group, both patients with breast cancer and patients with benign breast diseases had higher CA-125
Zhang et al. (2014) [24]	-	China	Female breast cancer patients with brain metastases Age group 26–79 years Diagnosis of breast cancer by biopsy	IV stage	166	Serum	Breast cancer patients with brain metastases’ CA-125 and CA-153 express levels are correlated to their clinicopathologic feature
Zhang et al. (2013) [25]	Follow-up study	China	-	-	65	Serum	CA-125 in the recurrence group was higher than in the non-recurrence group
Yuan et al. (2009) [26]	Retrospective study	China	Female breast cancer without the use of adjuvant chemotherapy or neoadju and trastuzumab Sufficient sample to investigate biological factors	-	274	Serum	No significant correlation exists between CA-125 and HER2 status in invasive ductal breast cancer patients
Tang et al. (2021) [27]	Retrospective observational study	China	Women with breast cancer Postmenopausal status Primary breast cancer	Primary ocular malignancies or benign tumors without pathology reports	865	Serum	Our investigation suggests that CA-125, remarkably predicts intraocular metastases in postmenopausal breast cancer as risk factors, and the combination of CA-125 and CA 15-3 shows considerable diagnostic value
Wang et al. (2015) [28]	-	China	-	Poor general conditions Failure to tolerate the side effects of the chemotherapeutic agent(s) Malignant disease (other than breast cancer) in the past 5 years Immunological disease	348	Serum	CA-125 has little clinical significance in predicting neoadjuvant treatment response in locally advanced breast cancer
Winden et al. (2012) [29]	Nested case-control study	Netherlands	Breast cancer within three years of entering the cohort Menopausal women	Diabetes Present smokers Using oral contraceptives or menopausal hormone therapy	Breast cancer: 68 Healthy controls: 68	Serum	The panel of selected tumor markers cannot be used for the diagnosis of early breast cancer.
López-Jornet et al. (2021) [30]	Cross-sectional study	Spain	Women with breast cancer Age over 18 years Control group: women were matched with breast cancer patients in terms of age, body weight, and body mass index (BMI) No history of malignancy	Fixed orthodontics Drug treatments associated with gingival overgrowth (nifedipine, cyclosporine, and phenytoin) Psychomotor disorders	Breast cancer patients: 91 Controls: 60	Saliva	The salivary biomarkers CA-125 appear to be promising tools in the diagnosis of breast cancer
Nazmeen et al. (2017) [31]	Cross-sectional study	India	-	-	-	Serum/tissue	CA-125 is a predictive marker in ovarian/breast carcinoma depending on the disease’s nature/stages
Luan et al. (2021) [32]	Retrospective case-control study	China	Female patients Between 18 and 75 years No malignancy within 5 years before entering the study Detection of circulating tumor cells (CTC) and tumor marker tests before initiation of any treatment	Pregnancy or breastfeeding at the time of CTC detection or serum tumor marker tests The time between CTC detection and serum tumor marker tests over 48 hours Extreme values of test results of CTC or serum tumor markers (1,000 times higher than average)	Breast cancer: 141 Control women: 71	Serum	CA-125 was not considered a biomarker for breast cancer
Luo et al. (2023) [33]	Retrospective observational study	China	Breast cancer: other malignant tumors and gynecological diseases or benign breast lesions in patients Breast cancer patients in line with the guidelines and norms for diagnosis and treatment of breast cancer Benign lesions: patients diagnosed with pathologically benign breast lesions (such as breast fibroadenoma)	Breast cancer: other malignant tumors and gynecological diseases Severe diseases, such as those of the liver, kidney, or heart Pregnant or lactating women Benign lesions: patients with malignant tumors or gynecological diseases Patients with severe diseases, such as those of the liver, kidney, or heart Pregnant or lactating women	Breast cancer: 108 Benign lesions: 77	Serum	Combinations of Alpha-fetoprotein (AFP) + CA153 + CA-125 have high accuracy (80.25%) in the screening and diagnosis of female breast cancer
Feng et al. (2020) [34]	-	China	Confirmation of breast cancer using pathology 20–60 years old Examination of the patient within 3 years after the radical operation Smooth operation and dissection of lymph nodes No severe complications	Abnormal PET-CT imaging caused by abscesses and active infection History of thyroid diseases, fractures, osteoarthritis, and osteoporosis; patients with other organ metastases Using drugs affecting bone metabolism Hormone therapy Autoimmune diseases Severe heart, liver, and kidney disease Other malignant tumors Severe infectious diseases	Bone metastasis: 60 Non-bone metastasis: 58	Serum	CA-125 may be involved in the occurrence and progression of bone metastasis of breast cancer
Geng et al. (2022) [35]	Prospective study	China	Confirmation of breast cancer using pathology Not using neoadjuvant chemotherapy Clinically n0 and some regions n1 Taking blood samples within 3 days before surgery	-	705	Serum	CEA, CA-125, CA153, tumor size, vascular invasion, calcification, and tumor grade were independent prognostic factors for positive lymph node metastasis
Kosmas et al. (2005) [36]	-	Greece	Confirmation of breast cancer in terms of histology Treatment with different chemotherapy regimens Tumor markers measurable in cerebrospinal fluid (CSF)	-	5	CSF and serum	CSF tumor marker evaluation may provide a reliable means and surrogate end-points for monitoring the response of carcinomatous meningitis to treatment
Tornos et al. (2005) [37]	-	USA	Confirmation of the diagnosis of metastatic breast carcinoma	-	Ovarian carcinoma: 42 Breast carcinoma: 36 Metastasis: 39	-	The presence of immunoreactivity for wt1 and CA-125 in a carcinoma involving the ovary strongly favors a primary lesion
Moritani et al. (2008) [38]	Case-control study	Japan	-	-	Breast cancer: 37 Genital organ cancer: 23	-	CA-125 is not a sufficient marker to differentiate Invasive micropapillary carcinoma of the breast from serous papillary adenocarcinoma
Dong et al. (2015) [39]	Retrospective study	China	-	-	26	Serum	No significant difference in the CEA and CA-125 serum levels between confirmed positive and confirmed negative PET/CT groups was found
Lin et al. (2018) [40]	Cohort study	China	-	Past radiotherapy or chemotherapy Non-cooperation in follow-up	486	Serum	Higher levels of preoperative serum tumor markers, such as CA-125, could represent tumor burden and have been suggested to be independent risk factors for the prognosis of breast cancer
Li et al. (2019) [41]	Retrospective analysis	China	Invasive breast cancer patients Age ≤ 40 years old treated during that period	Patients without follow-up CEA, CA-125, or other necessary data could not be extracted Neoadjuvant chemotherapy before surgery Metastasis at the time of diagnosis Previous or coexisting cancers Severe disease that influences patients’ survival	576	Serum	Preoperative serum CA-125 levels could be the independent prognostic factors for overall survival
Li et al. (2017) [42]	-	China	-	Infections Diabetes Encyesis Other reasons that might cause a high serum level	168	Serum	Serum CA-125 levels after the operation have certain instructional significance for the prognosis of breast cancer patients
Fan et al. (2022) [43]	Retrospective study	China	Invasive breast cancer Mastectomy or breast surgery and armpit lymph nodes Absence of distant metastasis Standard postoperative systemic treatment and regular follow-up examinations No other malignancy at the first visit	Less than 18 years of age Lack of standard systemic treatment after surgery Bilateral breast cancer Distant metastasis or occurrence of primary malignancy elsewhere Preoperative blood loss Postoperative infections	190	Serum	The tumor marker of CA-125 has potential prognostic value for breast carcinoma
Lou et al. (2020) [44]	Retrospective analysis	China	Triple-negative breast cancer Age 18–75 years Receiving radical mastectomy and regular chemotherapy after the operation	Incomplete clinical data Serious diseases, heart and kidney diseases, and other diseases Absence of mental illness or brain disease Absence of other cancers or non-primary breast cancer	Triple-negative breast cancer: 107 Non-triple-negative breast cancer: 235	Serum	The combination of serum CA-125, CA153, and CEA has a certain value in the diagnosis of triple-negative breast cancer, and high levels of CA-125 and CA153 after the operation in triple-negative breast cancer Patients indicate poor prognosis
Li et al. (2020) [45]	Cohort study	China	Pathologically confirmed breast cancer	Patients with stage IV at diagnosis Preoperative metastasis Receiving neoadjuvant chemotherapy	10,836	Serum	CA-125 is directly associated with aggressive clinicopathological characteristics

### Diagnosis

In recent years, there has been an increasing acknowledgment of the significance of using tumor markers to assist in detecting and diagnosing breast cancer. Researchers have become more and more attracted to the ease and trustworthiness of these markers as important resources in the field. While some experts argue against the use of tumor markers for diagnosing breast cancer [29], others assert that CA-125 can serve as a reliable aid in the diagnostic process [30, 31].

Though CA-125 is not considered a definitive diagnostic tool for breast cancer, a study conducted by Luan et al. [32] suggests that it can play a role in confirming the diagnosis in specific cases. This specific tumor marker is commonly used in conjunction with CEA and CA15-3 for diagnosing breast cancer, even though it has lower sensitivity and greater specificity.

Before surgery, the serum CA-125 levels differ among patients with breast cancer and those with benign conditions. Nevertheless, certain cancer patients exhibit tumor marker levels that exceed the defined threshold, indicating that these markers may not be very dependable in precisely identifying breast cancer [14]. In a study by Luo et al. [33], it was found that although the CA-125 marker increases in patients with benign disease and breast cancer, there is a statistically significant difference between the two groups. The combination of AFP, CEA, and CA153, as well as AFP and CA153 with CA-125, is reported to have the highest accuracy rate of 80.25% for breast cancer screening [32]. While tumor markers can aid in monitoring metastatic and symptomatic disease, their role in diagnosing breast cancer needs further clarification.

#### Diagnosis of the type and stage of cancer

CA-125 is utilized to determine the stage of the disease [31]. While this tumor marker can indicate disease progression, it cannot identify primary lesions [17]. Patients with late-stage tumors show elevated CA-125 levels in contrast to individuals with early-stage tumors, indicating the effectiveness of this tumor marker for tracking advanced stages [14]. The CA-125 level in stage IV breast cancer is elevated to over 90% [16]. In triple-negative tumors, CA-125 more commonly increases [19], and an elevation in CA-125 is linked to the status of lymph nodes [23]. There is no correlation between tumor marker levels, including CA-125, and specific tumor types, such as human epidermal growth factor receptor 2 (HER2)-positive or HER2-negative [26]. CA-125 levels have been found to be correlated with tumor progression [34].

#### Early detection of metastasis

The timely identification of metastasis is crucial for facilitating prompt treatment and efficient handling of the disease in its early phases. In a study conducted by Norum et al. [16], it was found that CA-125 serves as an important tumor marker that shows an initial increase during metastasis, potentially indicating the location of the metastatic spread. This elevation in CA-125 levels has been associated with larger tumor size (exceeding 5 cm) and the presence of lymph node metastasis [14]. Furthermore, research by Geng et al. [35] identified several independent prognostic factors for positive lymph nodes, including platelet numbers 1 and 2, CEA levels, tumor size, vascular invasion, calcification, and tumor grade. These results offer valuable insights into the possible indicators and prognostic factors linked to metastasis, aiding in the comprehension and treatment of the disease [35]. Moreover, an elevation in CA-125 is also observed in peritoneal metastases [19]. CA-125 levels serve as a risk indicator for bone metastasis. Feng et al. [34] reported higher CA-125 levels in patients with bone metastasis compared to those with non-bone metastasis and benign lesions. The reported sensitivity and specificity for predicting bone metastasis are 61.6% and 83.36%, respectively [18]. In breast cancer patients with brain metastasis, the level of CA-125 appears to be associated with both the clinical and pathological status of the individual [24], and the CA-125 level can be utilized to monitor carcinoma meningitis [36]. In research conducted by Tornos et al. [37], the goal was to differentiate primary ovarian cancer from breast cancer that has metastasized to the ovary. The research indicated that the presence of WT1 and CA-125 immunoreactivity in ovarian cancer suggests the probability of it being a primary tumor. Usually, ovarian carcinomas show both of these markers, whereas metastatic breast carcinomas to the ovary frequently do not. Moritani questioned the efficacy of CA-125 in differentiating between papillary serous adenocarcinoma of genital organs and breast cancer [38]. Similarly, Dong’s research concluded that the presence of CA-125 did not have significant diagnostic value in predicting metastasis [39].

#### Early diagnosis of recurrence

The primary objective of monitoring breast cancer patients is to promptly identify any new instances of primary breast cancer or recurrence. This early detection enables timely medical intervention and ultimately leads to improved survival rates for patients [46]. The sensitivity of tumor markers is influenced by the location of disease recurrence [47]. Although CA-125 levels in patients with cancer recurrence are higher than in cases of initial diagnosis [20] and in most cases of cancer recurrence [21], their serial measurement can suggest recurrence, and their low sensitivity is less effective in the follow-up process. According to research conducted by Gioia et al. [15], CA-125 has a sensitivity of 29.8% for detecting recurrence and a specificity of 100%. However, when combined with CEA and CA15-3 tumor markers, the sensitivity increases without affecting specificity. Therefore, the recommended approach for monitoring breast cancer patients post-treatment is to measure CA-125 along with other tumor markers. The study by Einama et al. [48] found that co-expression of mesothelin and CA-125 predicts a low survival rate without recurrence. In a different study by Dong et al. [39], it was found that CA-125 did not hold any diagnostic value when it came to predicting recurrence. Therefore, an increase in CA-125 may not always be the initial indication of a recurrence.

#### Diagnosis of treatment efficacy

Tracking how a cancer patient is responding to treatment is crucial for their care. According to a study conducted by Zhang and colleagues [17], monitoring the levels of CA-125 in the patient's blood confirms whether the neoadjuvant treatment is effectively working.

#### Prognosis and survival rate

CA-125 can predict prognosis and disease burden in breast cancer patients [22, 40–42]. The preoperative CA-125 test is a useful predictor for breast carcinoma [43]. The survival rate was lower in patients with high CA-125 levels compared to those with normal levels [19, 22]. When the level of CA-125 is less than 35 U/mL, patients tend to have a more favorable prognosis [24]. Overall survival was lower in triple-negative breast cancer patients with increased CA-125 compared to those without [44]. Following a multivariate Cox proportional hazard regression analysis, it was found that multiple variables served as independent prognostic indicators for overall survival. The variables comprise a familial history of breast cancer, tumor site, quantity of positive lymph nodes, histological grade, serum CEA, along with plate numbers 1 and 2 [40]. However, Li et al. [45] stated there is no relationship between the increase in CA-125 and the outcome.

## Discussion

Breast cancer is the most common type of cancer in women and the leading cause of death from malignant tumors [49–51]. The incidence of breast cancer worldwide has been increasing by 0.5% annually [51–53]. The decline in breast cancer deaths is due to early detection and prompt treatment [54].

It is crucial to emphasize the importance of early and accurate diagnosis in minimizing mortality, increasing survival rates, and enhancing the quality of life for individuals with breast cancer [55, 56]. Clinical studies have demonstrated that mammography can lower breast cancer mortality by 20% [57, 58]. The late detection of breast cancer continues to pose a significant challenge in developing nations. Research indicates that a delay of more than three months between symptom identification and treatment can result in advanced stages of the disease. This underscores the critical nature of early detection and prompt intervention in mitigating the impact of breast cancer [57].

While mammography is a widely recognized method for diagnosing breast cancer, its high cost and limited accessibility can create challenges in certain communities [59]. Utilizing molecular and less invasive methods has the potential to mitigate disparities in breast cancer diagnosis and detection [60, 61]. Thus, this study aims to examine the effectiveness of CA-125 in the management of breast cancer.

Mucin 16 (MUC16) has abundant glycosyl sites at the molecular level and participates in various molecular pathways. MUC16 is not expressed in normal epithelial cells, but it is present in metaplasia and neoplasia, such as ovarian, breast, pancreas, and colon malignancies. When cells lose their polarity and become cancerous, MUC16 is overexpressed and releases more of the extracellular domain, i.e., CA-125, into the serum, therefore contributing to cancer development [62]. CA125, which is a member of the mucin family of glycoproteins, promotes cancer cell growth and suppresses antitumor immunity [63]. This heavily glycosylated mucin protein [64] is produced by the epithelium derived from the celom and covers the peritoneum, pleura, and pericardium [65]. Under normal conditions, only small amounts of CA-125 are present in the bloodstream. However, during inflammatory reactions, elevated levels of CA-125 can affect physiological states [64]. Also, menstruation [66], pregnancy [67], liver disease [68], and nephrotic syndrome [69] may also increase CA-125 levels.

The detection method of CA-125 is simple, quick, and less invasive [69]. This tumor marker plays an important role in the diagnosis and prognosis of breast cancer [70]. Increased levels of CA-125 are seen in prostate cancer [71], lung carcinoma [72], colorectal carcinoma [73], ovarian epithelial cancers [74], endometrial carcinoma [75], cervical carcinoma [76], pancreatic carcinoma [77], and lymphoma [78]. Given that a high level of CA-125 is not specific to breast cancer and lacks supporting evidence, it is important to investigate other conditions that could elevate CA-125 levels. However, it is also crucial not to overlook the potential benefits of analyzing CA-125 in the context of breast cancer. CA-125, a novel biomarker in breast cancer diagnosis and prognosis, can be detected in nipple discharge, serum, and milk [79, 80].

Our research indicates that CA-125 can play a role in identifying primary tumors, metastasis, and recurrence, as well as determining the type and stage of breast cancer. The reliability of CA-125 as a diagnostic marker for distinguishing between benign and malignant primary breast tumors has been reported inconsistently. While some studies do not consider this biomarker to be sufficiently sensitive for diagnosing primary and malignant breast tumors [14, 29], others have demonstrated its diagnostic value either alone [30, 31] or in combination with other biomarkers such as AFP, CA-153, and CEA [32, 33]. According to a study, single tumor indicators have limitations when used as methods for assessing prognosis in breast cancer [79]. The sensitivities of the single tumor indicators were comparable: CEA at 7.18%, CA125 at 4.89%, CA15-3 at 7.47%, and TAP at 4.89% [80]. A study examined CA15-3, CEA, CA-125, and CA19-9 in 164 patients with metastatic breast cancer and found that CEA had the highest sensitivity, while CA-125 had the highest specificity when using just one marker for the diagnosis of metastatic breast cancer [81]. When considering the combinations of TAP + CEA + CA-125, TAP + CEA + CA15-3, TAP + CA-125 + CA15-3, and TAP + CEA + CA-125 + CA15-3, the sensitivities increased to 16.67%, 17.82%, 16.38%, and 21.84%, respectively. The specificities for these combinations were 93.49%, 97.70%, 93.87%, and 92.72% [80]. CA-125 is thought to originate from proliferating mesothelial cells rather than solely from cancer cells. It can be found in a wide range of both malignant and benign effusions. Therefore, these markers should not be used alone for the diagnosis of breast cancer in patients who have serous effusions [82].

In terms of diagnosing breast cancer metastasis, there are conflicting findings, but most studies have indicated its high diagnostic value [14, 18, 19, 24, 34–37]. Additionally, the three tumor markers CA-125, MUC1, and CEA are seen as complementary in the diagnosis of primary metastases [16]. Discrepancies in reported results can be attributed to variations in study design, participant demographics, and specific research objectives, such as impact on diagnosis, screening, survival, and prognosis. Therefore, investigating the sensitivity and specificity of the CA-125 biomarker (alone or in combination with other biomarkers) in screening, detecting recurrence, and predicting breast cancer prognosis needs further investigation.

In the early 1980s, CA-125 was initially utilized as a diagnostic marker for ovarian cancer. However, it was found that CA-125 levels can also be elevated in various physiological and pathological conditions such as pregnancy, menstruation, and endometriosis. This led to challenges in using CA-125 as a standalone marker for early-stage ovarian cancer diagnosis due to the high occurrence of false positives and negatives. As a result, additional biomarkers have been integrated with CA-125 for more accurate ovarian cancer diagnosis [71, 80, 81].

The findings of the current research demonstrate that CA-125 exhibits greater diagnostic efficacy in advanced stages of breast cancer compared to early-stage tumors. Elevated CA-125 levels can serve as a prognostic indicator for the disease. Additionally, the expression of CA-125 is influenced by the biological characteristics of different molecular subtypes of breast cancer. Patients with triple-negative tumors display significantly higher levels of CA-125 compared to those with luminal A, luminal B, and HER2/neu tumors [14].

The CA-125 biomarker has shown promise in identifying the recurrence of breast cancer either on its own or when combined with other biomarkers [15, 20, 21, 48]. However, it does not have a predictive role in recurrence [39]. Given the potential side effects of frequent radiographic imaging [83, 84], biomarkers offer a safer alternative for monitoring and providing post-surgery care for cancer patients [85].

The usefulness of CA-125 as a prognostic marker in breast cancer is still debated. Although certain studies indicate that increased CA-125 levels are associated with advanced disease and particular molecular subtypes [86, 87], others contend that it does not provide sufficient sensitivity and specificity for accurate prognosis [88].

The study’s reliance on English-language articles may have restricted the results by excluding valuable data from other languages. Additionally, due to the limited number of studies on the subject, a quality assessment of the articles was not conducted, and all studies meeting the inclusion criteria were included. The majority of research examined in this systematic review was carried out in China, which may limit the broader applicability of the findings. Also, the variations in the number of participants involved in the studies could have potentially impacted the findings. However, the comprehensive review of the role of CA-125 in breast cancer screening and diagnosis stands out as a strong aspect of the study.

The role of CA-125 as a biomarker for early detection, staging, and monitoring of recurrence and metastasis in breast cancer is still uncertain and needs additional research.

## Abbreviations

CA-125: cancer antigen 125
CEA: carcinoembryonic antigen
HER2: human epidermal growth factor receptor 2
MUC16: mucin 16
PRISMA: Preferred Reporting Items for Systematic Reviews and Meta-Analyses

## Declarations

### Availability of data and materials

The primary data for this systematic review were sourced online from databases listed in the methods. Referenced articles are accessible on PubMed/MEDLINE, Scopus, and Web of Science databases. Additional supporting data are available from the corresponding author upon request.

### Copyright

© The Author(s) 2025.
